# Forecasting call and chat volumes at online helplines for mental health

**DOI:** 10.1186/s12889-023-15887-2

**Published:** 2023-05-27

**Authors:** Tim Rens de Boer, Saskia Mérelle, Sandjai Bhulai, Renske Gilissen, Rob van der Mei

**Affiliations:** 1 Centrum Wiskunde & Informatica, Amsterdam, the Netherlands; 2 113 Suicide Prevention, Amsterdam, the Netherlands; 3 Vrije Universiteit Amsterdam, Amsterdam, the Netherlands

**Keywords:** Suicide prevention helpline, Health, Data analytics, Forecasting, Machine learning

## Abstract

**Background:**

Each year, many help seekers in need contact health helplines for mental support. It is crucial that they receive support immediately, and that waiting times are minimal. In order to minimize delay, helplines must have adequate staffing levels, especially during peak hours. This has raised the need for means to predict the call and chat volumes ahead of time accurately. Motivated by this, in this paper, we analyze real-life data to develop models for accurately forecasting call volumes, for both phone and chat conversations for online mental health support.

**Methods:**

This research was conducted on real call and chat data (adequately anonymized) provided by 113 Suicide Prevention (Over ons | 113 Zelfmoordpreventie) (throughout referred to as ‘113’), the online helpline for suicide prevention in the Netherlands. Chat and phone call data were analyzed to better understand the important factors that influence the call arrival process. These factors were then used as input to several Machine Learning (ML) models to forecast the number of call and chat arrivals. Next to that, senior counselors of the helpline completed a web-based questionnaire after each shift to assess their perception of the workload.

**Results:**

This study has led to several remarkable and key insights. First, the most important factors that determine the call volumes for the helpline are the *trend*, and *weekly* and *daily* cyclic patterns (cycles), while monthly and yearly cycles were found to be non-significant predictors for the number of phone and chat conversations. Second, *media events* that were included in this study only have limited—and only short-term—impact on the *call volumes.* Third, so-called (S)ARIMA models are shown to lead to the most accurate prediction in the case of short-term forecasting, while simple linear models work best for long-term forecasting. Fourth, questionnaires filled in by senior counselors show that the experienced workload is mainly correlated to the number of chat conversations compared to phone calls.

**Conclusion:**

(S)ARIMA models can best be used to forecast the number of daily chats and phone calls with a MAPE of less than 10 in short-term forecasting. These models perform better than other models showing that the number of arrivals depends on historical data. These forecasts can be used as support for planning the number of counselors needed. Furthermore, the questionnaire data show that the workload experienced by senior counselors is more dependent on the number of chat arrivals and less on the number of available agents, showing the value of insight into the arrival process of conversations.

## Background

Many countries have helplines to support people struggling with mental health problems, such as suicidal thoughts [2]. These helplines provide immediate and anonymous support, often free of cost, to improve mental health and prevent suicides [3]. The Netherlands has multiple helplines, like the listen helpline (Dutch: *de Luisterlijn*) [4], the helpline for children (Dutch: *de Kindertelefoon*) [5], and 113 Suicide Prevention, which is the helpline for suicide prevention in the Netherlands, providing telephone-call as well as chat support [6]. This paper focuses on the helpline of 113 Suicide Prevention, but the methodology and results also provide important insights for other helplines. In the Netherlands alone, on average five persons die each day by suicide [7], and worldwide more than 700,000 people annually [8]. Suicide is a global mental health phenomenon. 113 Suicide Prevention has the mission that no one should die alone and in despair of suicide and to break the taboo around suicide. This national suicide prevention center started as 113Online founded by Jan Mokkenstorm on 7 October 2009. Help seekers with suicidal thoughts or their family and friends can contact 113 round-the-clock anonymously, either by telephone or chat. Besides mental health services, 113 also provides training services, leads the National Suicide Prevention Agenda [9] and has a research department. The organization is subsidized by the Ministry of Health, Welfare and Sport. During and after COVID-19, 113 saw increased chat and phone call arrivals, showing the importance of helplines during crisis situations [10]. Unpaid *volunteers* and paid *professionals* assist help seekers at 113. It is crucial to gain insight into the arrival process of these help seekers to help these people as well as possible because these insights can contribute to good predictions of call volumes, and hence adequate staffing levels, resulting in lower waiting times and a higher number of help seekers helped.

In our study, various factors were considered vital for the number of help seekers per day, such as the historic trend, daily, weekly, monthly, and yearly patterns, and the effect of large news items or events discussed in various media forms. Whitley et al.[11] and Niederkrotenthaler et al. [12] researched the influence of media events on suicides and found that the number of suicides increased *after* the suicide of a well-known celebrity.

Research has been done on forecasting call volumes at helplines (e.g., [13]). Gijo et al. showed the added benefit of using (S)ARIMA models to forecast call volumes in emergency services [14]. (S)ARIMA models can identify possible trends and cycles, Gijo et al. show that forecasting can be done effectively using historical data only. In contrast, research on helplines for mental health is often focused on the conversation topics or the types of callers, rather than on call volumes and waiting times. For example, Salmi et al. showed the change in conversation topics during COVID-19 [15]. Grigorash et al. have studied the caller type of mental health helplines [16]. In this context, the present paper aims to fill the gap between these studies by *combining* a forecasting approach mostly seen in general call centers on the one hand with the specifics of the mental health helpline context where media events might affect the demand on the other hand.

This study aims to test and compare different forecasting models on the anonymous call-volume data provided by 113. Assumptions about the helpline are validated using data analysis, especially a possible trend, cycle effects (over different time scales), and exogenous factors, such as the effect of media events. A *cycle* is defined as a seasonal effect, which repeats over time on a yearly, monthly, weekly, or even daily basis. *Conversely, the trend* shows the general tendency of the data to increase or decrease during a longer period. More specifically, we address the following hypotheses:

- 1. Weekly, daily, and yearly cycles are important for predicting the number of arrivals.
- 2. The number of incoming phone calls and chats increases during and after a large media event (such as the suicide of a celebrity).
- 3. Accurately forecasting the call volumes is possible using models that use historical data with possible exogenous factors. The historical data is used to incorporate possible cycles and trends, while exogenous factors are added to use the possible effect of media events.
- 4. The workload that counselors experience increases when the average waiting time for phone or chat is higher than usual. This applies to situations where there are more help seekers than counselors can help or more help seekers than expected.

## Methods

### Dataset

The data provided by 113 consists of (anonymized) call and chat conversations ranging from 2017 until 2021 and contains around 250,000 chats and 175,000 telephone calls. This dataset is time-stamped per conversation and can therefore be used separately to test hourly and/or daily forecasting for chat and telephone. The size of the dataset also makes it possible to determine effect cycles as well as identify a possible trend. The details of the number of chat and telephone conversations can be found in Table 1. Each call or chat record contains the following fields: the *contact id*, an *initial contact id*, (the *channel* (telephone or chat), the *arrival time*, the *time entering the queue*, the *accept time*, the *disconnect time*, the *completion time*, the *switch count*, and finally, the *agent* that handled the call or chat. Here, the contact id is used to identify the conversation, where contact id is the same as the initial contact id if the conversation is not forwarded, agents can forward conversations if the help seeker requires more or other help. The switch count also identifies the number of times a call has been forwarded, so the switch count would be one in the case of contact id equal to initial contact id. This paper focuses on conversations where the contact id is equal to the initial contact id. The switch count increases with one, with each conversation sent through. The data contains four timestamps, the previously mentioned arrival, queue enter, accept, and disconnect time. Help seekers arrive at 113 at the arrival time. Help seekers can be classified into two groups based on their means of communication, help seekers that call 113 (the so-called *phone callers*) and help seekers that use the chat function of 113 to communicate with 113 (the so-called *chatters*). Phone callers first have to listen to a phone tape and fill in a questionnaire, chatters are also required first to fill in a questionnaire. After the help seeker has filled in all questions, he or she enters the queue. The time it takes to fill in the questions is the so-called *pre-queue duration*. The chatter/caller waits until a counselor is available, and then the conversation is accepted. After finishing the conversation, the help seeker and counselor are disconnected, and finally, the agents have to fill in a *wrap-up form*. The wrap-up form is a form for the agent to evaluate the conversation and the help seeker, recording the conversation topic, for example, and if this person has called or chatted with 113 before; the time it takes to fill in this form is called the *wrap-up duration*.

**Table 1:** Number of chat and telephone arrivals per year

Year	Number of chat arrivals	Percentage of chat arrivals of total	Number of telephone arrivals	Percentage of telephone arrivals of total	Total number of arrivals
2017	40,036	69%	18,078	31%	58,114
2018	48,114	65%	26,410	35%	74,524
2019	57,335	58%	41,993	42%	99,328
2020	65,945	55%	53,651	45%	119,596
2021	81,582	55%	67,286	45%	148,868

### Data preprocessing

Not all data were useful in its original form. Therefore, we identified missing values in the data and determined whether any imputations were required. Missing values are handled differently based on context: a missing value in waiting time often meant that the help seeker abandoned the queue. In some cases could also have been due to the help seeker being accepted before being queued. These values were filled in based on these conditions. Finally, the data were aggregated to obtain call and chat volumes per day and hour. We had two days for which the call and chat volumes were both zero, probably due to a technical issue. These two volumes were estimated using linear interpolation.

### Forecasting models

The following models were used to forecast day volumes: ARIMA, SARIMA, Linear Regression, LSTM, and various baseline models. These models were chosen to represent different approaches for forecasting and could incorporate the different aspects we hypothesized as important.

(Seasonal) Autoregressive Integrated Moving Average (shortly, (S)ARIMA) models are well-known time series models [14] used for forecasting. ARIMA uses previously measured values for forecasting future values. SARIMA is similar to ARIMA, but here a *seasonal component* is added; see [17] for an overview of (S)ARIMA models. The parameters for ARIMA and SARIMA are both determined using AutoARIMA [18]. AutoARIMA is an R-method that determines the best parameters based on the Akaike Information Criterion (AIC), which is an estimator of the prediction error. Linear regression is a simple machine learning (ML) approach and was used to fit a linear trend with a weekly effect on the data. These models fit a linear relation between various factors and the outcome, in this case, the number of arrivals. In formula form, this model looks as follows:

$${\varvec{F}}={\varvec{X}}{\varvec{\beta}}+{\varvec{\varepsilon}}$$

where ***F*** is a vector containing the forecasts, ***X*** is a vector containing the input variables, ***β*** is the vector containing parameters, and finally, **ε** is noise.

The Long Short Term Memory (LSTM) model is a more sophisticated ML model used for forecasting in time series and is a special kind of Recurrent Neural Network (RNN). Lastly, these models are compared to various baseline models: the forecast of day *i* is the measurement of day *i*-7 or *i*-56, calculated as follows:

$${F}_{i}={A}_{i-7}$$

For Baselines 1 and 2, the following is used:

$${F}_{i}={A}_{i-56}$$

where ${F}_{i}$ is the forecast for day *i* and ${A}_{i}$ is the actual value of day *i*. These baselines correspond to using the actual number of phone calls and chats from one week ago (7 days) or 8 weeks (56 days). The models are compared based on the Mean Absolute Percentage Error (MAPE), defined as follows:

$${\text{MAPE}}=\frac{1}{n}\sum_{i=1}^{n}\left|\frac{{F}_{i}-{A}_{i}}{{A}_{i}}\right| \times 100\%$$

where *n* is the number of forecasts.

### Questionnaire

Next, a questionnaire was given to senior counselors to determine *when* and *why* they experienced a high workload. This process consists of a questionnaire based on a quick scan for workload and adapted to the situation of the helpline. Senior counselors were asked to fill in a questionnaire after each shift [19]. Here, we examine whether the workload of the senior counselors is related to the number of calls or chats, and are so able, together with the forecasts, to understand adequate staffing levels. Data collection took place from 16 February to 30 April 2022. Table 2 gives an overview of the 10-item questionnaire that uses a 5-point Likert scale [20]. We transformed all questions such that a score of “5” indicates a high workload and a score of “1” indicates a low workload. Questionnaires that reported technical problems were excluded from analyses since high experienced workload can then be related to the technical issues. We can identify whether there were any technical issues based on positive answers to Question 7.

**Table 2:** Questions of the questionnaire

Number	Question	Answers
1	Did you have many tasks to do during the shift?	Very busy, busy, neither, unbusy, very unbusy
2	Did you have to work hard to do everything?	Always, often, usually, sometimes, never
3	Did you have to rush during your shift?	Always, often, usually, sometimes, never
4	Was there a large backlog, many missed conversations or a high waiting time?	Always, often, usually, sometimes, never
5	Did you have issues with the pace?	Always, often, usually, sometimes, never
6	Could you show interest for colleagues?	Always, often, usually, sometimes, never
7	Were there any technical issues during your shift?	Always, often, usually, sometimes, never
8	Could you forward the chats in triage?	Very well, well, neither, bad, very bad
9	Did you have energy left at the end of your shift?	Very much, much, neither, little, very little
10	Were there enough counselors besides interns?	Yes, neutral, no

The sum scale that represents the counselor’s experienced workload is calculated by summing all questions, except Question 7, since this question is used to filter out questionnaires with technical issues.^1^ This sum scale was then compared to features of the objective workload captured by the data. These variables are: the number of chats and phone calls during the shift, mean waiting time of chats and phone calls, missed percentage of chats, and phone calls. The Cronbach’s alpha of the questionnaire was 0.72 [21], which tells us that using the questionnaire in its current form is acceptable. Pearson’s correlation coefficient was then used to measure the strength of the relationship, where 0 means no relation and 1 or -1 means a perfect correlation [22]. P-values < 0.05 were considered statistically significant.

## Results

### Trend

**Fig. 1:**
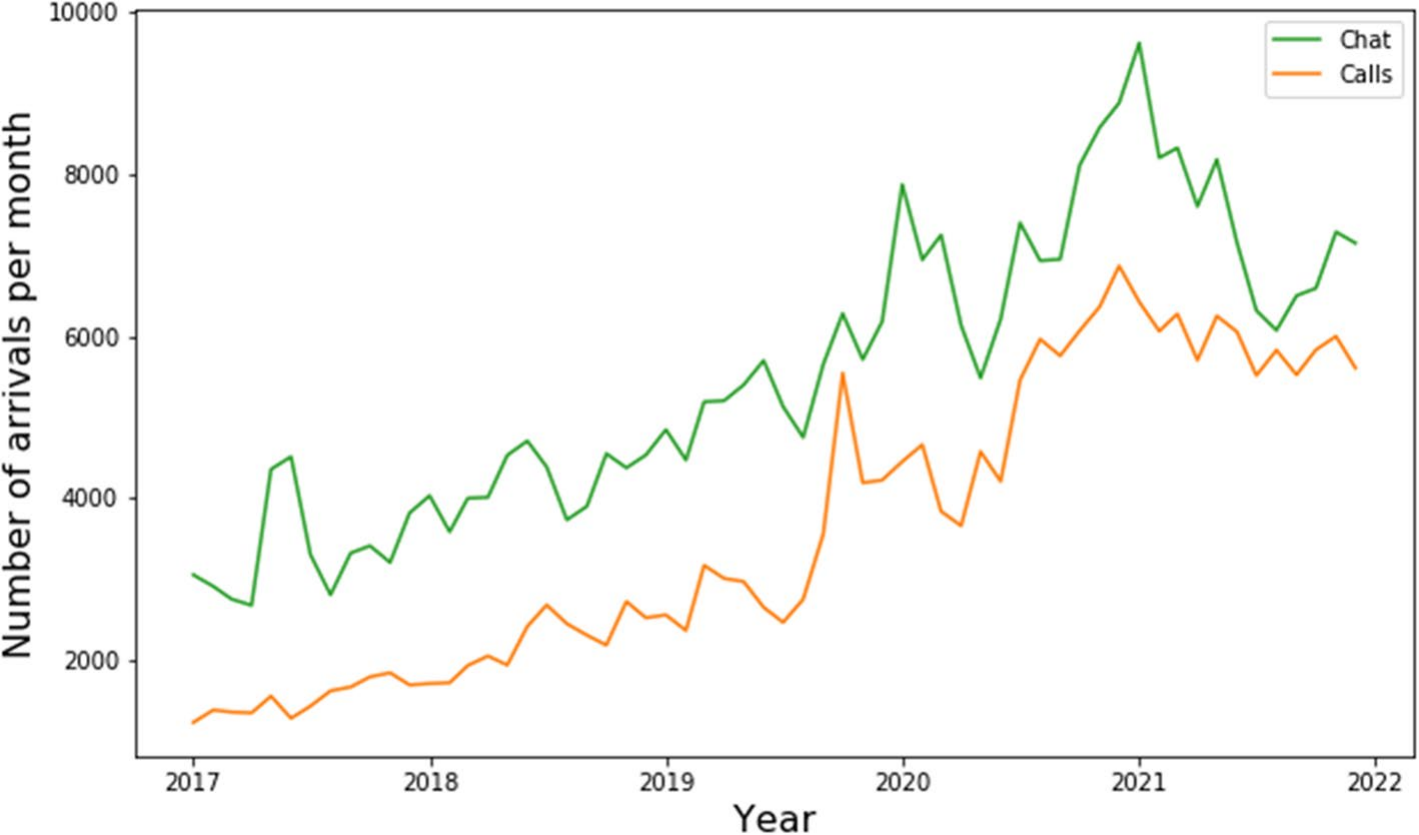
The number of arrivals per month

The number of telephone and chat conversations shows an increasing trend over the years (see Fig. 1 below). This can also be seen in Table 1, where the number of phone and chat conversations more than doubled in the period from 2017 until 2021. In 2017, on average, around 110 chats and 50 telephone calls were arriving daily. In 2021 we observed 224 chats and 184 telephone calls per day.

### Weekly and daily patterns

**Fig. 2:**
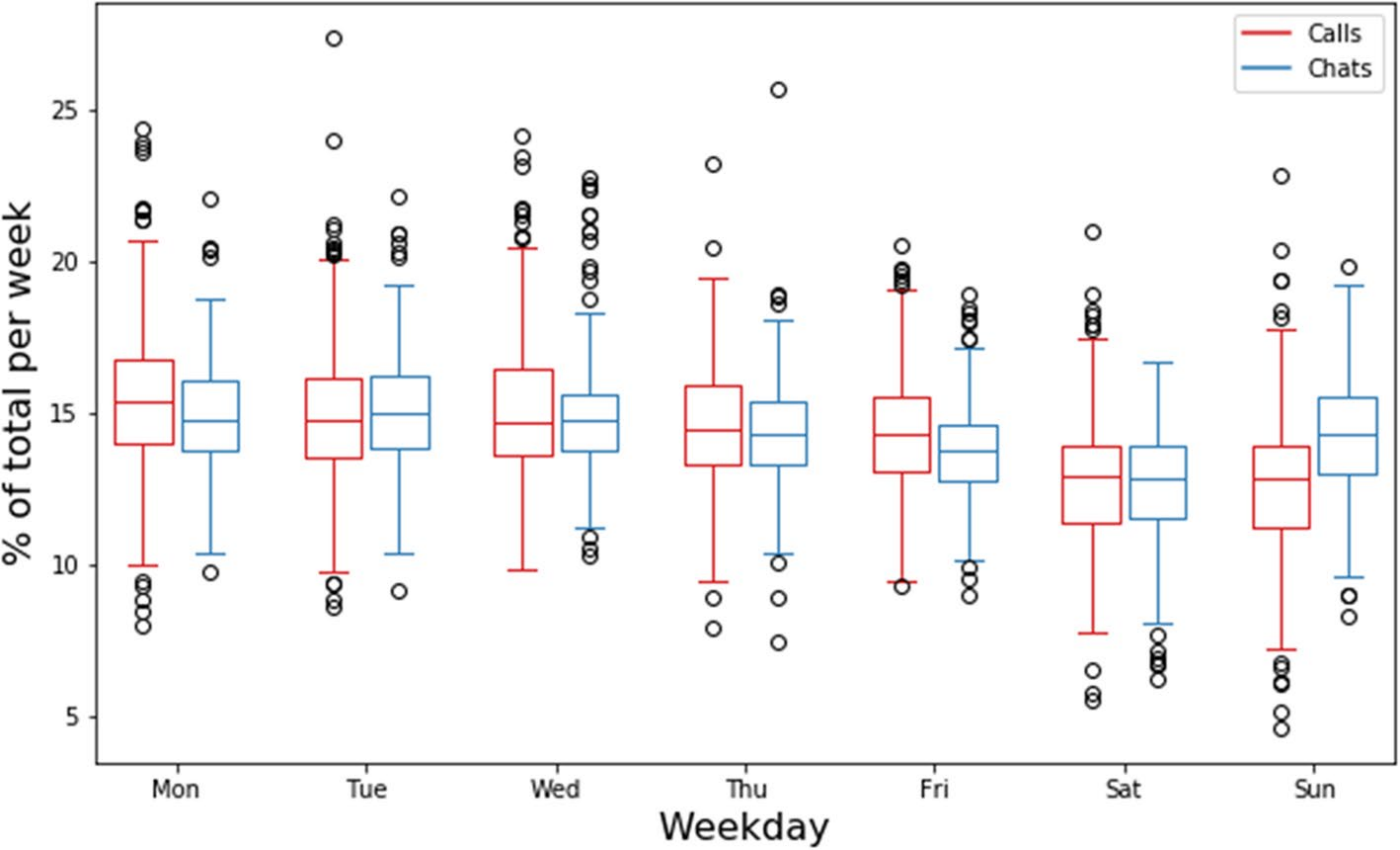
Weekly cycle of phone calls and chats

First, the weekly pattern was determined. This was done by determining the distribution of arrivals over the different weekdays. As shown in Fig. 2, the distribution of arrivals over the week from Monday until Friday for the telephone is similar around 15%, with a drop in the weekend to around 12.7%. We see a slightly different cycle for the chats: the number of chat arrivals is similar for days from Monday until Thursday, with a drop on Friday and a larger drop on Saturday, followed by an increase on Sunday.

**Fig. 3:**
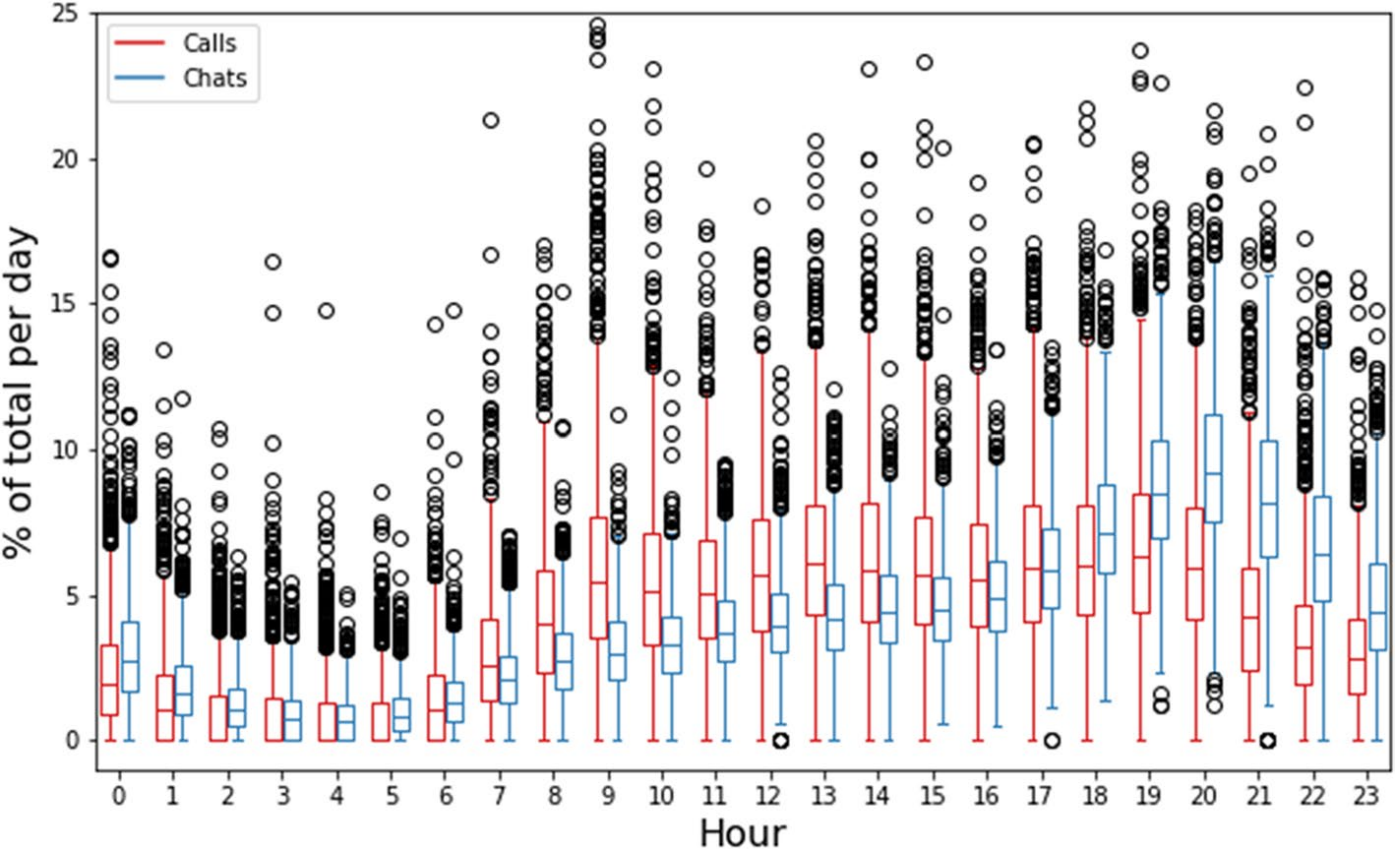
Daily cycle of phone calls and chats

Next, the daily cycles were examined, similarly to determining the weekly cycles. The daily cycles can be found in Fig. 3, telephone and chat arrivals both show a dip in the early morning from 1 AM until 5 AM. The number of telephone arrivals is similar during the period from 9 AM till 8 PM, while chat arrivals show a clear peak in the evening around 8 PM.

Both daily and weekly cycles show the effects of these cycles for forecasting the number of arrivals. Besides the importance of the different cycles, it also shows the importance of forecasting chats and telephone conversations as two separate arrival processes, since they follow different cycles. *Yearly* and *monthly cycles* were also analyzed. However, both were found to *not significantly vary over time*, possibly due to limited available data in the case of yearly cycles.

### Media events

**Fig. 4:**
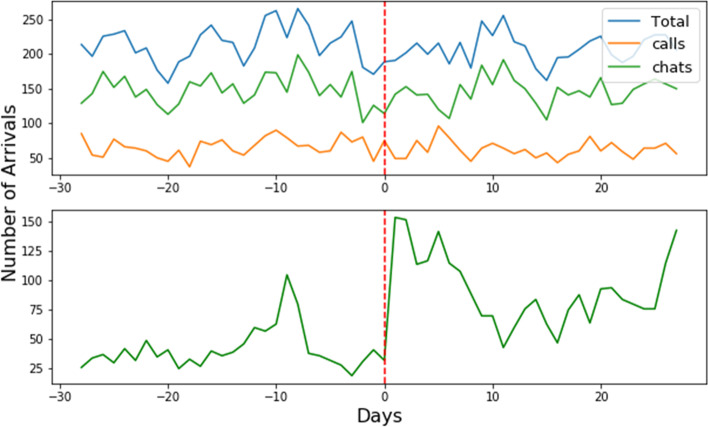
Suicide of an internationally well-known artist on day 0 (upper graph) and suicide of a nationally well-known author on day 0 (lower graph)

**Fig. 5:**
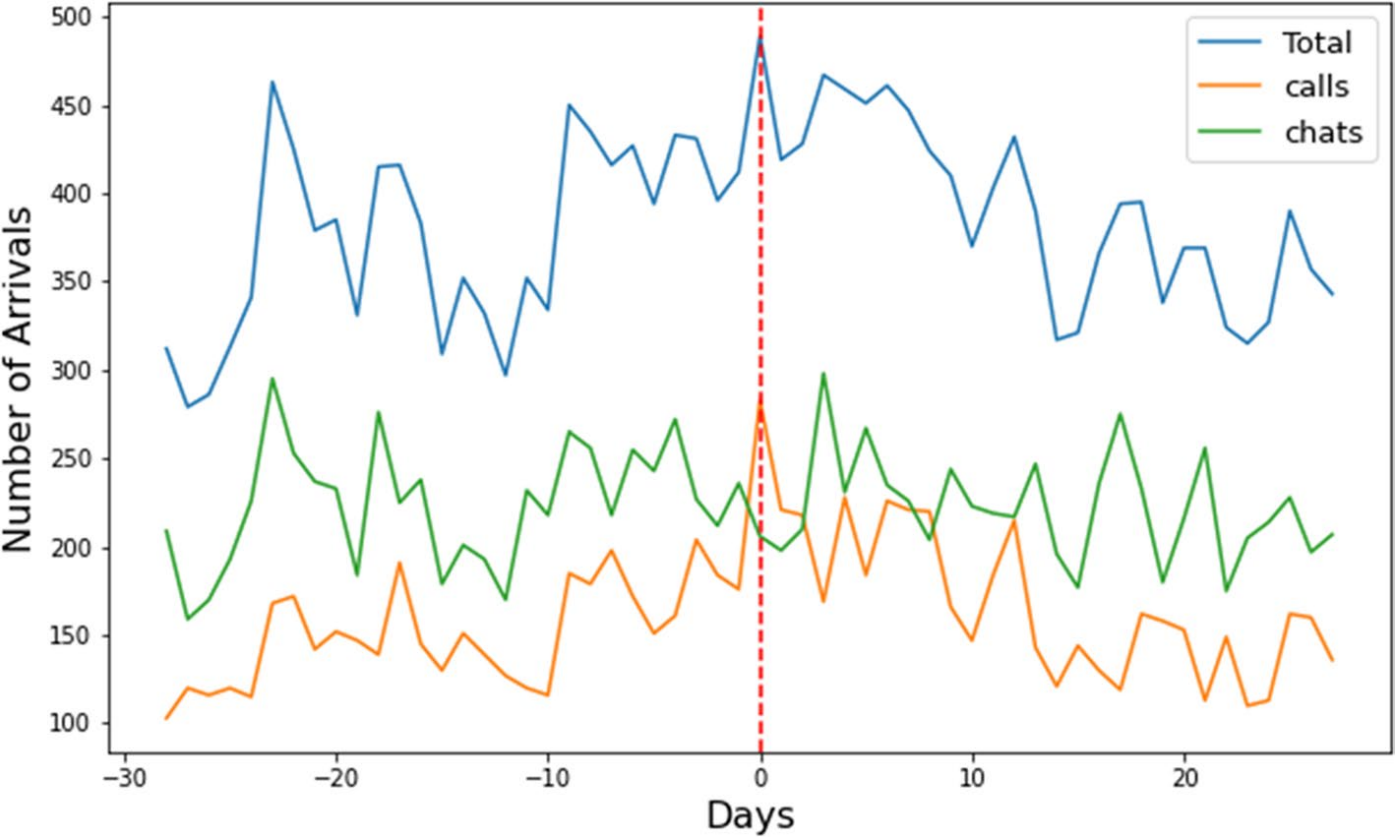
113 receiving media attention due to political news, peak on day 0

To assess whether celebrity suicides might influence the *number of call arrivals* at the helpline, the periods *before* and *after* celebrity suicides were analyzed, and briefly outlined below. In the period studied, one celebrity in the Netherlands died by suicide. Figure 4 (lower graph) shows the effect of the suicide of a well-known Dutch author on the number of chat arrivals. In this period, only the data of chats are available. It is clear that the news significantly affected the number of chats, especially in the week after the news broke. Figure 4 (upper graph) also shows the absence of the effect the suicide of an internationally well-known artist had on the arrivals at the helpline. Lastly, Fig. 5 shows the effect national political news had on the arrivals, as an example of the effect of media events other than the suicide of well-known persons. We observe that in most cases the effects of these events were limited, or only short-term (one or two days). Figure 4 (lower graph) shows that there are events that have a larger or long-term effect, but most events did not have this large or long-term effect. Together with the fact that this type of event cannot be predicted ahead of time, we chose not to include these events in the forecasting models.

### Forecast results

The forecast analyses were done separately for chats and phone calls. The error (in terms of the MAPE) for each model and each time window can be seen in Tables 3 and 4 for chat and telephone, respectively. The lowest MAPE, meaning the most accurate forecast. In both cases, the ARIMA and SARIMA models perform similarly and best in the case of short-term forecasting, five weeks or less for telephone, and seven weeks or less for chats. After these time windows, in both cases, the simple models perform the best for long-term forecasting, which can be seen in Tables 3 and 4, where the MAPE of the simple model (12.80 for chat and 15.01 for phone) is less than that of the (S)ARIMA models. Most remarkably, both (S)ARIMA and the simple models have a lower MAPE than the baseline model and the LSTM model, which performs even worse.

**Table 3:** Error term of the chat forecast models

Days ahead	ARIMA	SARIMA	Baseline 1	Baseline 2	Simple model	LSTM
1	8.63	8.28	11.56	14.63	12.80	15.24
2	9.04	8.66	11.56	14.63	12.80	16.78
3	9.27	8.99	11.56	14.63	12.80	16.41
4	9.53	9.30	11.56	14.63	12.80	16.60
5	9.68	9.44	11.56	14.63	12.80	16.50
6	9.84	9.61	11.56	14.63	12.80	16.57
7	9.78	9.58	11.56	14.63	12.80	16.94
8	10.04	9.78		14.63	12.80	
9	10.31	9.89		14.63	12.80	
10	10.44	10.07		14.63	12.80	
11	10.46	10.09		14.63	12.80	
12	10.48	10.33		14.63	12.80	
13	10.42	10.37		14.63	12.80	
14	10.49	10.43		14.63	12.80	
15	10.54	10.39		14.63	12.80	
16	10.76	10.55		14.63	12.80	
17	10.87	10.68		14.63	12.80	
18	10.98	10.74		14.63	12.80	
19	11.14	11.01		14.63	12.80	
20	11.25	11.07		14.63	12.80	
21	11.27	11.24		14.63	12.80	
22	11.57	11.43		14.63	12.80	
23	11.55	11.48		14.63	12.80	
24	11.57	11.52		14.63	12.80	
25	11.61	11.73		14.63	12.80	
26	11.59	11.65		14.63	12.80	
27	11.45	11.53		14.63	12.80	
28	11.21	11.37		14.63	12.80	
29	11.38	11.44		14.63	12.80	
30	11.46	11.49		14.63	12.80	
31	11.27	11.33		14.63	12.80	
32	11.13	11.41		14.63	12.80	
33	11.08	11.39		14.63	12.80	
34	11.26	11.65		14.63	12.80	
35	11.24	11.62		14.63	12.80	
36	11.24	11.59		14.63	12.80	
37	11.47	11.90		14.63	12.80	
38	11.55	11.93		14.63	12.80	
39	11.59	12.08		14.63	12.80	
40	11.83	12.20		14.63	12.80	
41	11.97	12.42		14.63	12.80	
42	12.25	12.67		14.63	12.80	
43	12.56	12.84		14.63	12.80	
44	12.60	12.96		14.63	12.80	
45	12.74	13.10		14.63	12.80	
46	12.61	13.00		14.63	12.80	
47	12.66	13.04		14.63	12.80	
48	12.66	13.19		14.63	12.80	
49	12.82	13.28		14.63	12.80	
50	12.85	13.12		14.63	12.80	
51	12.86	13.15		14.63	12.80	
52	12.99	13.21		14.63	12.80	
53	13.01	13.34		14.63	12.80	
54	13.18	13.50		14.63	12.80	
55	13.32	13.83		14.63	12.80	
56	13.29	13.74		14.63	12.80

**Table 4:** Error term of the telephone forecast models

Days ahead	ARIMA	SARIMA	Baseline 1	Baseline 2	Simple model	LSTM
1	11.30	10.79	15.73	19.08	15.01	18.85
2	11.71	11.26	15.73	19.08	15.01	19.13
3	11.77	11.24	15.73	19.08	15.01	19.71
4	12.02	11.54	15.73	19.08	15.01	19.48
5	12.31	11.82	15.73	19.08	15.01	20.21
6	12.36	11.96	15.73	19.08	15.01	18.8
7	12.35	12.03	15.73	19.08	15.01	19.34
8	12.68	12.14		19.08	15.01	
9	12.85	12.27		19.08	15.01	
10	12.69	12.35		19.08	15.01	
11	12.47	12.19		19.08	15.01	
12	12.65	12.21		19.08	15.01	
13	12.76	12.47		19.08	15.01	
14	12.62	12.51		19.08	15.01	
15	12.88	12.61		19.08	15.01	
16	13.05	12.56		19.08	15.01	
17	13.13	12.74		19.08	15.01	
18	13.11	12.78		19.08	15.01	
19	13.33	13.03		19.08	15.01	
20	13.19	13.09		19.08	15.01	
21	13.37	13.40		19.08	15.01	
22	13.72	13.64		19.08	15.01	
23	13.89	13.85		19.08	15.01	
24	13.75	13.83		19.08	15.01	
25	13.76	13.76		19.08	15.01	
26	13.81	13.73		19.08	15.01	
27	13.85	13.89		19.08	15.01	
28	13.88	13.97		19.08	15.01	
29	14.04	14.12		19.08	15.01	
30	14.30	14.22		19.08	15.01	
31	14.28	14.10		19.08	15.01	
32	14.19	14.11		19.08	15.01	
33	14.45	14.34		19.08	15.01	
34	14.44	14.48		19.08	15.01	
35	14.49	14.72		19.08	15.01	
36	14.59	14.86		19.08	15.01	
37	14.80	14.97		19.08	15.01	
38	15.03	15.21		19.08	15.01	
39	15.50	15.57		19.08	15.01	
40	15.58	15.56		19.08	15.01	
41	15.98	16.11		19.08	15.01	
42	16.30	16.46		19.08	15.01	
43	16.55	16.63		19.08	15.01	
44	16.78	16.72		19.08	15.01	
45	16.83	16.88		19.08	15.01	
46	16.90	17.02		19.08	15.01	
47	16.86	17.05		19.08	15.01	
48	16.94	17.15		19.08	15.01	
49	17.02	17.30		19.08	15.01	
50	17.14	17.35		19.08	15.01	
51	17.08	17.22		19.08	15.01	
52	17.05	17.12		19.08	15.01	
53	17.28	17.27		19.08	15.01	
54	17.21	17.19		19.08	15.01	
55	17.27	17.20		19.08	15.01	
56	17.59	17.50		19.08	15.01	

**Fig. 6:**
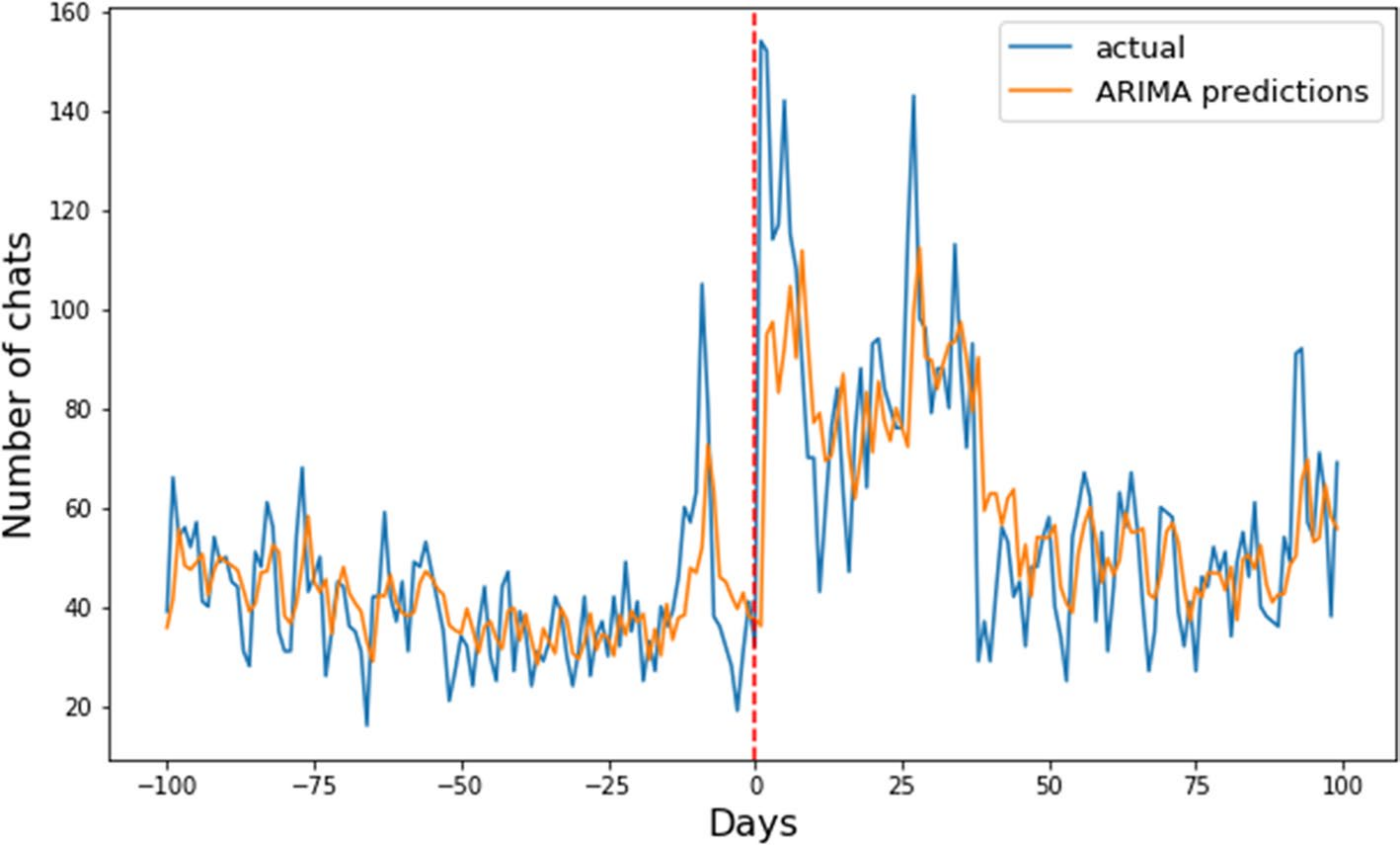
Chat predictions before, during, and after a media event with a large and long-lasting effect

A demo of how one-day ahead predictions using (S)ARIMA work can be seen below. Figure 6 shows that in the case of an event with a large effect the (S)ARIMA model can quickly adapt to the increase in the number of arrivals. The (S)ARIMA quickly adapts *without* explicitly giving the event or the *reason* for the increase. We observe that the predictions follow the waves of the arrivals and also adapt in cases of peaks and throughs.

### Experienced workload

The senior counselors filled in 88 questionnaires (*n* = 32 Day, *n* = 41 Evening, *n* = 13 Night), two were excluded from analyses since respondents reported technical problems during the shift. Descriptive statistics are given in Table 5. Most questions are filled in with a mean of around 3, except for Questions 5,6, and 10, which all have a mean below 2. These answers indicate that senior counselors, in general, experience fewer problems with the pace, can still show interest in their colleagues, and have enough counselors besides interns in the shift. In contrast, they experience more problems due to the multitude of tasks, which can be seen by the mean of Question 1, the highest mean score of all questions.

**Table 5:** Descriptive statistics of the useable questionnaires

Question	Mean	Standard deviation
1	3.03	0.82
2	2.99	0.86
3	2.28	1.01
4	2.52	0.94
5	1.85	0.77
6	1.98	0.53
8	2.52	1.06
9	2.99	0.79
10	1.91	0.79

**Fig. 7:**
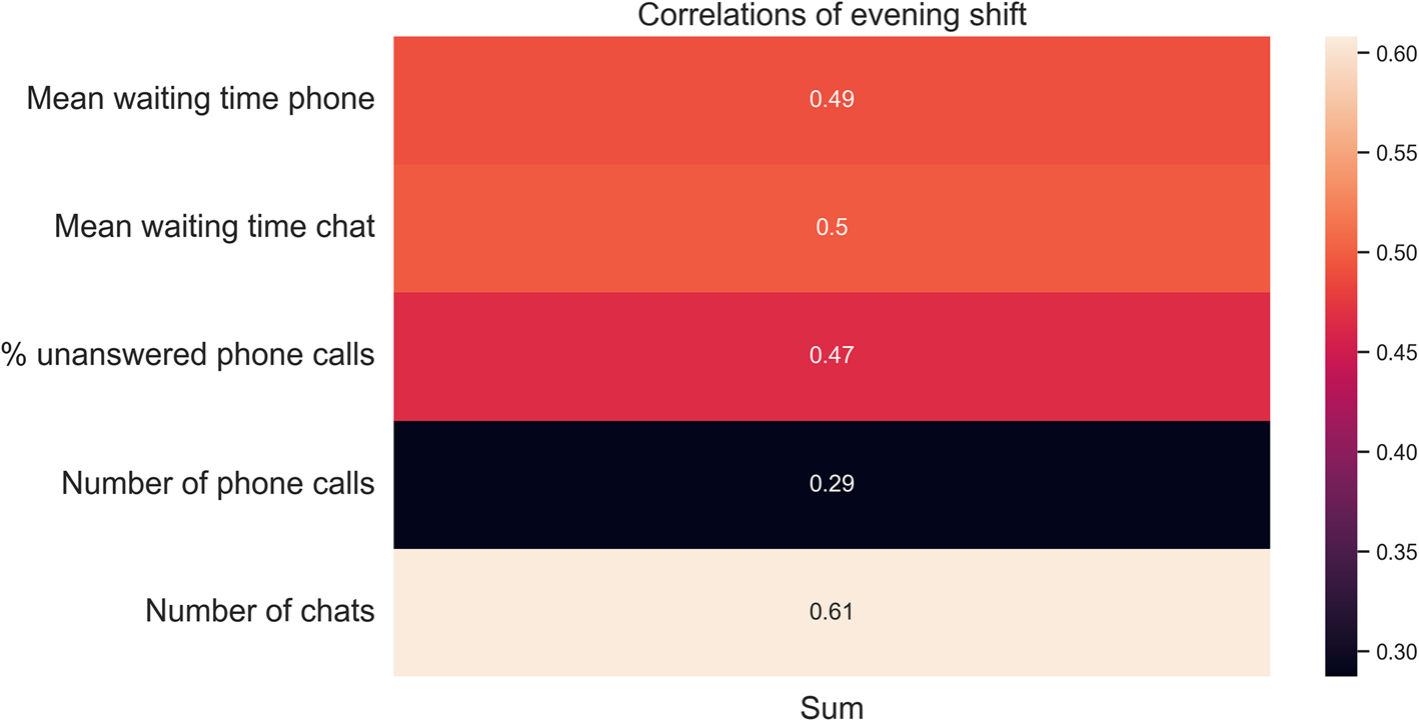
Correlations between the outcome variables and the questionnaires of the evening shift

Next, it was checked whether the questionnaire data contained some busy shifts; this was done by checking the number of arrivals of each shift. It is found that the questionnaires filled in by the evening shift contain the most variability of workload. The correlations found in the evening shift are presented in Fig. 7. We found that most correlations were significant, except for the correlation between the sum score and the number of phone calls (see Table 6), albeit moderately correlated (i.e., around or below 0.5). The two strongest relations are between the number of chats and the total sum score. The correlations of the percentage of unanswered chats are omitted since all chats were answered.

**Table 6:** Correlations between the outcome variables and the questionnaires of the evening shift

Outcome variable	Correlations with the sum score	*P*-value
Mean waiting time phone	0.49	0.001
Mean waiting time chat	0.50	0.001
% Unanswered phone calls	0.47	0.002
Number of phone calls	0.29	0.069
Number of chats	0.61	Smaller than 0.001

## Discussion

This paper aimed to shed light on the factors that determine call volumes at online mental health helplines. Based on real-life data from the helpline of 113 Suicide Prevention, we found that the following factors are dominant: *trend*, and *weekly* and *daily* cycles. The media events appear only to have a limited—or short-term—effect on the number of arrivals, contrary to the effect these kinds of events have on the number of suicides studied by Whitley et al. [10] and Niederkrotenthaler et al. [11]. To our knowledge, previous work primarily focuses on the different help-seeker *types* arriving at the helpline [16], but *not* on the *actual arrival process*. The insight that (S)ARIMA forecasts are most accurate shows that the arrivals at the helpline are mostly dependent on historical data and can be used at other helplines that handle (mental health) emergencies, which is comparable to what Gijo et al. found [14].

We also found that telephone forecasting can best be done with (*S)ARIMA models* for *short-term forecasting* (less than four weeks) and *linear regression* for *long-term* forecasting (more than four weeks). Chat forecasting can best be done by (S)ARIMA models for the whole test forecasting period of eight weeks or less ahead. Surprisingly, the (S)ARIMA models performed better than the LSTM models. However, it could be the case that the LSTM model can improve with more time and optimization. However, it is questionable if with more optimization and time the LSTM will perform better than the other models. The low MAPE of the (S)ARIMA models can be attributed to the workings of the models. These models are flexible. In case of an event with a large and long-lasting effect, the (S)ARIMA models can *quickly adapt* and include this increase or decrease in the forecasts. Overall, the rule holds that the forecasts lose accuracy when forecasting further in the future.

The results of the questionnaire show that the experienced workload of the counselors is mostly related to the number of chats during a shift. Surprisingly, the experienced workload seems to have a weaker relationship with the workload of the phone calls. Both are crucial insights into the causes of experienced workload, which was previously done for only volunteers [23]. However, it should be noted that the results of the questionnaire showed that, on average, senior counselors do not experience a high workload or seem able to work with a high workload. A higher variability in experienced workload during shifts is needed to determine the relationship between call volumes and waiting times more precisely.

## Limitations

Most of the limitations encountered with this research can be attributed to the availability or quality of the data. Yearly cycles could not accurately be measured, since the data consisted of five years of chats and phone calls. However, recently 113 has seen enormous growth in the number of chat and phone calls, making it difficult to measure the yearly cycle if one is present accurately.

Media events were considered to influence the number of arrivals. The data shows that suicides of *nationally* well-known celebrities might have more impact than those of *inter*nationally well-known persons. Luckily, the number of Dutch well-known persons that died by suicide is limited. Therefore, it is unknown whether a similar event nowadays would lead to a similar, smaller, or larger effect.

The found correlations with the questionnaire were all significant except for the correlation between the number of phone calls and the sum score. However, more data might be needed to say more about the significance of the correlations, given the sample size of 41 on which the calculations were made.

## Implications

One of the key implications considered is that the planning department of 113 Suicide Prevention can use the predictions provided by the forecasting models. The predictions offer the possibility to adjust the staffing and schedule. Staffing better fitting to the number of arrivals can relieve counselors and volunteers from stress and provides them with more time to cool down after a difficult conversation [23].

### Possibilities for future research

There are several directions for future research. First, we may extend the model to include different caller types of the helpline and determine the arrival processes per type. This could introduce a larger forecasting error but could provide more information on what type of help-seeker to expect and *when* and *how* these callers could best be helped.

The effect of the level of experience of counselors on the duration of the conversation is an interesting issue. Initial results suggest no significant correlation, but this could be investigated in more detail, making distinctions between functions and different levels of experience.

## Conclusion

The analysis of real-life data leads to new and important insights in forecasting the demand for online health support for mental health. The (S)ARIMA model forecasts have a MAPE of less than 10 in short-term forecasting, showing that the number of chats and telephones can be forecasted. The fact that (S)ARIMA models perform better than other models shows that the number of call and chat arrivals is more dependent on historical data, without explicitly giving data about media events. These forecasts can then be used in other processes, for example, to support the planning of counselors.

Furthermore, the results of the questionnaire show that the experienced workload of senior counselors is less dependent on the actual staffing and more on the number of chat arrivals. These results again show the importance of insight into the arrival process of the chat (and/or) telephone.

## Data Availability

The datasets used and/or analyzed during the current study are available from the corresponding author upon reasonable request.

## Abbreviations

(S)ARIMA: (Seasonal) Autoregressive Integrated Moving Average
LSTM: Long Short-Term Memory model
MAPE: Mean Absolute Percentage Error
AIC: Akaike Information Criterion
ML: Machine Learning

## Footnotes

1 The questions used in the sum scale do not include questions concerning only chats or phone calls, except for Question 8, which only concerns incoming chats. However, this question is included, since the question is about the process of forwarding chats after triage, chats after triage are handled by the same counselors that handle phone calls. The process of forwarding chats can therefore be obstructed by a large workload of phone calls. The results using the sum score excluding and including Question 8 and the resulting conclusions were similar. Therefore, we chose to use the collected data of this item.
